# Differences in *Toxoplasma gondii* distribution in different *muscle* and viscera of naturally infected sheep

**DOI:** 10.1371/journal.pone.0283867

**Published:** 2023-08-17

**Authors:** Xinlei Yan, Xindong Jin, Jialu Gao, Wenying Han, Yufei Sun, Xiuli Yu, Pufang Liu, Wenhui Guo, Jia Chen, Lin Su

**Affiliations:** Food Science and Engineering College of Inner Mongolia Agricultural University, 010018, Hohhot, China; Foshan University, CHINA

## Abstract

*Toxoplasma gondii* (*T*. *gondii*) is a zoonotic parasite that can cause serious pathology in intermediate hosts such as humans and animals. Eating undercooked or raw meat is the most important route of infection by *T*. *gondii*. Sheep are an important source of meat worldwide, and they are also susceptible to *T*. *gondii*. Mutton infected with *T*. *gondii* poses a serious threat to the food safety of consumers. At present, studies have mainly focused on the infection ratio of *T*. *gondii* in livestock; however, systematic studies have not been performed on differences in the distribution of this parasite in different muscle and viscera tissues of animals. In this study, the differences in the distribution of *T*. *gondii* in naturally infected Small-tailed Han sheep was studied. By amplifying the B1 gene of the parasite via real-time fluorescence quantification PCR (RT‒qPCR), we found that the parasite burden of *T*. *gondii* differed among different parts of the sheep, with the highest burden observed in the heart among the viscera and the external ridge among the *muscle*. The relative expression was ranked from high to low in our study as follows: heart, spleen, external ridge, tenderloin, lung, liver, kidney, neck meat, forelegs, cucumber strips, hind leg, lamb belly, and lamb chops. This study provided important guidance for monitoring the food safety of mutton products.

## 1. Introduction

*Toxoplasma gondii* (*T*. *gondii*) is a globally prevalent opportunistic pathogenic protozoan that exclusively parasitizes the nucleated cells of warm blooded animals and can cause serious harm to humans and animals [1,2]. It is one of the most geographically widespread parasites in the world and shows high diversity in its infecting host range [3]. Previous studies have demonstrated that the disease might affect a third of humans and revealed that the positive rate of the *T*. *gondii* antibody in China is approximately 10%-30% [4].

*T*. *gondii* infections are spread by a number of routes, and the ingestion of meat, fruit, vegetables, and even water containing cysts or oocysts is the main method of horizontal transmission of *T*. *gondii* [5]. Eating undercooked or raw meat contaminated with *T*. *gondii* cysts is the most important route of infection [6]. Meanwhile, transmission through the placenta can lead to fetal infection [7]. In Europe, 30%-63% of infections in pregnant women were attributed to meat intake [8]. According to the European Food Safety Authority, meat-borne transmission accounts for approximately 60% of *Toxoplasma* infections, and the main source is contaminated mutton, beef and pork [9].

In humans, the hazards caused by this protozoan mainly include miscarriage, stillbirth, organ inflammation, nerve center disorders, etc. People with normal immune function are generally asymptomatic after acquiring a small amount of *T*. *gondii*, although when a large number of *T*. *gondii* cysts or oocysts are ingested, the hosts will enter an acute infection stage and show clinical symptoms, such as fever, maculopapular rash, muscle and joint pain, and inflammation of various organs [10]. For pregnant women, infection with *T*. *gondii* in early pregnancy could lead to abortion, stillbirth, malformation and other adverse pregnancy outcomes. In addition, *T*. *gondii* can also secrete its own virulence protein, thus causing strong immune pathological damage to the placenta [11].

Livestock are susceptible to *Toxoplasma* infection, and many reports have investigated *T*. *gondii* infection in livestock worldwide [12]. Unfortunately, most of these studies have performed serological investigations, with few reports focusing on the distribution of the parasite in edible parts of livestock [13]. Mutton is one of the most important sources of meat worldwide; however, mutton consumption presents a high risk of infection with *T*. *gondii*. Although many studies have performed serological investigations of this protozoan as previously indicated [14], differences in the distribution of *T*. *gondii* in the edible parts of sheep have not been systematically studied. Therefore, in this study, we systematically studied the differences in the distribution of *T*. *gondii* in different *muscle* and viscera of naturally infected small-tailed Han sheep. The findings are of great significance for improving meat food safety and can also provide important guiding significance for monitoring the safety of the mutton product market. In addition, the results may provide helpful insights to aid consumers in selecting appropriate cooking methods for different parts of sheep.

## 2. Materials and methods

### 2.1 Serum samples

Blood samples were collected from small-tailed Han sheep maintained on a ranch in Hohhot, Inner Mongolia, China, to investigate the presence of serum antibodies against *T*. *gondii*. Blood samples were collected into a centrifuge tube(15ml) from the jugular vein of a total of 115 sheep (approximately 50% male and female and 8–12 months old). These centrifuge tubes filled with blood samples were quickly frozen in a local freezer and brought back to the laboratory in an incubator. Each of the blood samples was centrifuged at 3000 rpm for 10 min, and serum samples were separated and stored at -20°C until further analysis.

### 2.2 Determination of antibodies against *T*. *gondii*

Antibodies against *T*. *gondii* from serum samples were detected by an indirect enzyme-linked immunosorbent assay (ELISA) test using a commercially available kit (CK-DN74810, 96T; Quanzhou Ruixin Biotechnology Co., Ltd) according to the protocol described by the manufacturer.

### 2.3 Meat samples

A total of 7 serologically positive sheep weighing approximately 35 kg were selected for slaughter. The protocols were approved by the Experimental Animal Welfare Ethics Committee of Inner Mongolia Agricultural University (No: NND2021072). *muscle* and viscera were sampled according to the sheep anatomy map after slaughter. The sampled *muscle* (300–500 g) included the external ridge, tenderloin, neck meat, forelegs, cucumber strips, hind leg, lamb belly, lamb chops. The sampled viscera included the heart, liver, spleen, lung, and kidney.

### 2.4 DNA extraction

DNA extraction from samples was performed using a Tissue DNA Extraction Kit (DP304; Tiangen Biotechnology Co., Ltd.) according to the protocol described by the manufacturer.

### 2.5 PCR

The *T*. *gondii* B1 gene in the heart was amplified from 7 sheep, and the PCR products were analyzed by electrophoresis on a 2.5% agarose gel at a voltage of 120 V for approximately 20 min. PCR products identified as positive were stored at 4°C.

The following primers were used: B1 F: 5’-GGAACTGCATCCGTTCATGAG-3’, B1 R: 5’-TCTTTAAAGCGTTCGTGGTC-3’ [15]. The amplification system included 9 μL ddH_2_O, 12.5 μL 2×Taq PCR Master Mix, 0.5 μL B1-F, 0.5 μL B1-R, and 2.5 μL DNA. The amplification conditions were as follows: predenaturation at 94°C for 3 min followed by cycling, denaturation at 94°C for 30 s, annealing at 55°C for 45 s, extension at 72°C for 10 s, a total of 30 cycles, and a final extension at 72°C for 7 min.

### 2.6 RT‒qPCR

RT‒qPCR assays of the samples were performed using SuperReal PreMix (FP314) from Tiangen Biotechnology Co., Ltd. RT‒qPCR amplification was performed on all sampled parts of the sheep with positive PCR test results. The prepared plasmids with different concentration gradients can be amplified simultaneously. The amplification system included 6 μL ddH_2_O, 10 μL SYBR Green Master Mix, 1 μL B1-F, 1 μL B1-R, and 2 μL DNA. The amplification conditions were as follows: predenaturation at 95°C for 5 min followed by cycling and denaturation at 95°C for 10 s and annealing at 62°C for 30 s, for a total of 40 cycles.

### 2.7 Bioassays

Female BALB/c mice at 6 weeks old and weighing 15–18 g were purchased from SPF (Beijing) Biotechnology Co., Ltd. The mice were given food and water *ad libitum* and housed under a 12 h light/dark cycle. The animal protocols were reviewed and approved by the Inner Mongolia Agricultural University Laboratory Animal Welfare and Animal Experimental Ethical Inspection Committee (NND2021069). All experiments were performed according to the relevant guidelines and regulations. Fifty grams of sample was minced and digested in an acid pepsin solution (5.2 g pepsin (P7000-25 G; Sigma), 10 g NaCl and 14 mL HCl (pH 1.1)) for 1 h at 37°C under constant shaking at 60 rpm. The digested suspension was neutralized with a 1 N NaHCO_3_ solution at pH 8.3, filtered through 3 layers of gauze, and centrifuged at 1200×g for 10 min. Pellets were washed 3 times in PBS and resuspended in 5 mL of PBS with antibiotic (1000 U penicillin and 100 mg streptomycin per mL). 1 mL of the pellet suspension was inoculated intraperitoneally into Balb/c mice. The mice were kept for 6 weeks and then killed by cervical dislocation, and brain samples were then collected and stored at -80°C for subsequent detection of *T*. *gondii* by RT‒qPCR as previously described.

### 2.8 Statistical analysis

The Step One^TM^ Plus RT‒qPCR instrument was used in the experiment. After the reaction, the Ct value was recorded and the ΔΔCT value was calculated, with 2^-ΔΔCT^ indicating the result.

## 3. Results

A total of 115 serum samples were tested by ELISA. We found that the number of antibody-positive sheep was 37, and the overall infection rate was 32.2%. Seven sheep (C4, C10, C13, C16, C18, A8, and L1) with higher OD values were selected to obtain PCR samples. The B1 gene was amplified from the hearts of the 7 sheep, and the results showed that the *T*. *gondii* B1 gene could be amplified in 5 sheep, namely, L1, C4, C16, C18 and A8.

The relative expression of the B1 gene in the muscle and viscera samples from the 5 sheep was determined by RT‒qPCR, and then the viability of the parasites was bioassayed in the mice. As shown in Table 1, the relative expression of the B1 gene was highest in the heart and lowest in the lamb chops in each sheep, although the values differed widely among the 5 sheep. The relative expression of the B1 gene in the viscera was higher than that in all of the *muscle* except for the external ridge and tenderloin. The relative expression of *T*. *gondii* in the outer and inner ridges was the highest among the *muscle*, while that in the kidney was the lowest among the viscera. The relative expression level in the viscera was ranked (from high to low) as heart, spleen, lung, liver, kidney, while that in the muscle was ranked as external ridge, tenderloin, neck meat, forelegs, cucumber strips, hind leg, lamb belly, and lamb chops. Moreover, the bioassays results showed that nearly all tested edible parts contained infectious parasites, and 90% (4/5) to 100% (5/5) of the hearts were positive for *T*. *gondii* while only 0% (0/5) to 20 (1/5) of the lamb chops were positive for *T*. *gondii*.

**Table 1:** Differences in the distribution of *T*. *gondii* in different *muscle* and viscera of Small-tailed Han sheep.

sampling location	A8		L1		L9		C16		C18	
	2^-∆∆Ct^	Bio	2^-∆∆Ct^	Bio	2^-∆∆Ct^	Bio	2^-∆∆Ct^	Bio	2^-∆∆Ct^	Bio
heart	199.26	5/5	53.82	5/5	398.93	4/5	247.28	5/5	106.89	5/5
spleen	66.87	5/5	36.00	4/5	80.45	5/5	93.70	5/5	40.22	3/5
external ridge	56.74	4/5	28.44	4/5	66.26	5/5	62.25	4/5	35.26	4/5
tenderloin	58.10	4/5	28.05	3/5	57.68	4/5	62.25	2/5	34.54	4/5
lung	52.54	3/5	27.86	3/5	67.18	4/5	55.72	4/5	33.59	4/5
liver	49.44	4/5	27.10	4/5	66.72	4/5	45.57	3/5	32.45	3/5
kidney	36.58	3/5	26.35	3/5	50.56	2/5	44.32	2/5	31.12	2/5
neck meat	48.01	2/5	24.42	2/5	41.07	3/5	40.50	4/5	30.27	2/5
forelegs	27.98	2/5	24.08	2/5	38.32	4/5	36.76	3/5	23.10	1/5
cucumber strips	16.97	2/5	10.06	3/5	36.00	1/5	30.70	3/5	16.80	1/5
hind leg	25.43	1/5	10.41	1/5	31.56	2/5	27.86	2/5	15.24	0/5
lamb belly	7.95	1/5	0.74	0/5	8.40	1/5	2.87	0/5	2.39	0/5
lamb chops	1.00	0/5	1.00	0/5	1.00	0/5	1.00	0/5	1.00	1/5

Bio: *T*. *gondii* viability assays.

## 4. Discussion

In this study, the rate of positive antibodies in the sera of sheep was 32.2% according to ELISA, indicating that the sheep population had a higher chance of being infected with *T*. *gondii*. Sheep are strict herbivores, therefore, infection mainly occurs through the ingestion of *T*. *gondii* oocysts. Mild environmental conditions prolong the survival time of oocysts, thus causing a significant increase in the chance of infection during captivity [16]. Antibody detection is performed to identify the presence of specific anti-*Toxoplasma* antibodies, but the level of antibody test results is not directly related to the amount of *T*. *gondii* infection. Therefore, biomolecular detection is warranted.

In this study, we detected the differences in the distribution of *T*. *gondii* in different edible parts of small-tailed Han sheep by amplifying the B1 gene using RT‒qPCR. The results showed that *T*. *gondii* was distributed in viscera and various *muscle*, among which the heart had the highest parasitic burden. Moreover, the burden of *T*. *gondii* was generally higher in the viscera than the *muscle*. Related research has indicated that the distribution of the parasite in muscle samples is uneven, and even smaller portions (5 g and 10 g) of meat are likely to be carriers of *T*. *gondii* [17]. Recent research has shown that the P43 gene of *T*. *gondii* can be amplified in most edible parts of sheep using nested PCR [18], and a previous study isolated *T*. *gondii* from the chest, heart, diaphragm, waist, flat muscle and brain of pigs slaughtered 60 days after infection [19]. However, these studies only identified whether the edible parts of livestock contained this parasite. Our results systematically revealed the differences in the distribution of this protozoan in different parts of sheep for the first time. In this study, the distribution trend of *T*. *gondii* in each sheep was consistent, although differences were observed in the amount of *T*. *gondii* infection among the tested sheep. This phenomenon might be affected by many factors, such as individual variations in the animal and differences in the infection amount and parasite strain. Interestingly, the results showed that the burden of *T*. *gondii* was higher in the viscera than *muscle*. The reason might be the rich blood flow in these parts, which not only allowed *T*. *gondii* to quickly move and colonize through the blood or lymphatic system but also provided sufficient nutritional conditions for *T*. *gondii* replication. In this study, the head and hoof of sheep were not sampled, mainly because the head of sheep is not the main edible part and the content of *muscle* in the hoof is low [20]. Bioassays showed that live cysts existed in the tested parts even though the relative expression of the parasites was low. In our study, the higher the relative expression, the more infected the mice. The results showed that eating infected mutton has a high risk of infection with this parasite.

## 5. Conclusions

In this study, the differences in the distribution of *T*. *gondii* in different muscle and viscera tissues of sheep was studied systematically for the first time. The results showed that the distribution of *T*. *gondii* was different in the muscle and viscera tissues of tested sheep after natural infection with the parasite. Overall, this study revealed the risk of *T*. *gondii* infection in mutton and provided an important basis for the control of meat-borne parasites in food processing. Furthermore, the differences in the distribution of *T*. *gondii* in edible parts of other livestock and the mechanism of the differences in the distribution of this parasite in different parts of animals might be an interesting research field in the future.

## Supporting information

S1 File (XLSX)
